# Supplementary material for: Resolving the full spectrum of human genome variation using Linked-Reads
Source: Genome Res. 2019 Apr;29(4):635–45. doi: 10.1101/gr.234443.118 (PMC6442396; doi:10.1101/gr.234443.118)
Supplement: Supplemental Material [file supp_gr.234443.118_Supplemental_longranger-2.2.zip › longranger-2.2.tar.bz2!/lr2.2-public/lib/stan/cmdstan/stan/doc/api/html/json__parser_8hpp_source.html]

Stan: src/stan/io/json/json\_parser.hpp Source File


|  |  |
| --- | --- |
|  | Stan  2.5.0  probability, sampling & optimization |


- Main Page
- Related Pages
- Namespaces
- Classes
- Files

- File List
- File Members

All Classes Namespaces Files Functions Variables Typedefs Enumerations Enumerator Friends Macros Pages

- src
- stan
- io
- json

json\_parser.hpp

Go to the documentation of this file.

1 #ifndef STAN\_\_IO\_\_JSON\_\_JSON\_PARSER\_HPP

2 #define STAN\_\_IO\_\_JSON\_\_JSON\_PARSER\_HPP

3

4 #include <stdexcept>

5 #include <iostream>

6 #include <istream>

7 #include <sstream>

8 #include <string>

9

10 #include <boost/lexical\_cast.hpp>

11

12 #include <stan/io/json/json\_error.hpp>

13

14 namespace stan {

15

16  namespace json {

17

18  namespace {

19

20  const unsigned int MIN\_HIGH\_SURROGATE = 0xD800;

21  const unsigned int MAX\_HIGH\_SURROGATE = 0xDBFF;

22  const unsigned int MIN\_LOW\_SURROGATE = 0xDC00;

23  const unsigned int MAX\_LOW\_SURROGATE = 0xDFFF;

24  const unsigned int MIN\_SUPPLEMENTARY\_CODE\_POINT = 0x010000;

25

26  inline bool is\_high\_surrogate(unsigned int cp) {

27  return (cp >= MIN\_HIGH\_SURROGATE && cp <= MAX\_HIGH\_SURROGATE);

28  }

29

30  inline bool is\_low\_surrogate(unsigned int cp) {

31  return (cp >= MIN\_LOW\_SURROGATE && cp <= MAX\_LOW\_SURROGATE);

32  }

33

34  inline bool is\_whitespace(char c) {

35  return c == ' ' || c == '\n' || c == '\t' || c == '\r';

36  }

37

44  template <typename Handler, bool Validate\_UTF\_8>

45  class parser {

46

47  public:

48

49  parser(Handler& h,

50  std::istream& in)

51  : h\_(h),

52  in\_(in),

53  next\_char\_(0),

54  line\_(0),

55  column\_(0)

56  { }

57

58  ~parser() {

59  }

60

61  void parse() {

62  h\_.start\_text();

63  parse\_text();

64  h\_.end\_text();

65  }

66

67  private:

68

69  json\_error json\_exception(const std::string& msg) const {

70  std::stringstream ss;

71  ss << "Error in JSON parsing at"

72  << " line=" << line\_ << " column=" << column\_

73  << std::endl

74  << msg

75  << std::endl;

76  return json\_error(ss.str());

77  }

78

79  // JSON-text = object / array

80  void parse\_text() {

81  char c = get\_non\_ws\_char();

82  if (c == '{') { // begin-object

83  h\_.start\_object();

84  parse\_object\_members\_end\_object();

85  h\_.end\_object();

86  } else if (c == '[') { // begin-array

87  // array

88  h\_.start\_array();

89  parse\_array\_values\_end\_array();

90  h\_.end\_array();

91  } else {

92  throw json\_exception("expecting start of object ({) or array ([)");

93  }

94  }

95

96  // value = false / null / true / object / array / number / string

97  void parse\_value() {

98  // value

99  char c = get\_non\_ws\_char();

100  if (c == 'f') {

101  // false

102  parse\_false\_literal();

103  } else if (c == 'n') {

104  // null

105  parse\_null\_literal();

106  } else if (c == 't') {

107  // true

108  parse\_true\_literal();

109  } else if (c == '"') {

110  // string

111  h\_.string(parse\_string\_chars\_quotation\_mark());

112  } else if (c == '{' || c == '[') {

113  // object / array

114  unget\_char();

115  parse\_text();

116  } else if (c == '-' ||

117  (c >= '0' && c <= '9') ) {

118  unget\_char();

119  parse\_number();

120  } else {

121  throw json\_exception("illegal value, expecting object, array, number, string, or literal true/false/null");

122  }

123  }

124

125  void parse\_number() {

126  bool is\_positive = true;

127

128  std::stringstream ss;

129  char c = get\_non\_ws\_char();

130  // minus

131  if (c == '-') {

132  is\_positive = false;

133  ss << c;

134  c = get\_char();

135  }

136

137  // int

138  // zero / digit1-9

139  if (c < '0' || c > '9')

140  throw json\_exception("expecting int part of number");

141  ss << c;

142

143  // \*DIGIT

144  bool leading\_zero = (c == '0');

145  c = get\_char();

146  if (leading\_zero && (c == '0'))

147  throw json\_exception("zero padded numbers not allowed");

148  while (c >= '0' && c <= '9') {

149  ss << c;

150  c = get\_char();

151  }

152

153  // frac

154  bool is\_integer = true;

155  if (c == '.') {

156  is\_integer = false;

157  ss << '.';

158  c = get\_char();

159  if (c < '0' || c > '9')

160  throw json\_exception("expected digit after decimal");

161  ss << c;

162  c = get\_char();

163  while (c >= '0' && c <= '9') {

164  ss << c;

165  c = get\_char();

166  }

167  }

168

169  // exp

170  if (c == 'e' || c == 'E') {

171  is\_integer = false;

172  ss << c;

173  c = get\_char();

174  // minus / plus

175  if (c == '+' || c == '-') {

176  ss << c;

177  c = get\_char();

178  }

179  // 1\*DIGIT

180  if (c < '0' || c > '9')

181  throw json\_exception("expected digit after e/E");

182  while (c >= '0' && c <= '9') {

183  ss << c;

184  c = get\_char();

185  }

186  }

187  unget\_char();

188

189  if (is\_integer) {

190  if (is\_positive) {

191  unsigned long n;

192  try {

193  n = boost::lexical\_cast<unsigned long>(ss.str());

194  } catch (const boost::bad\_lexical\_cast & ) {

195  throw json\_exception("number exceeds integer range");

196  }

197  ss >> n;

198  h\_.number\_unsigned\_long(n);

199  } else {

200  long n;

201  try {

202  n = boost::lexical\_cast<unsigned long>(ss.str());

203  } catch (const boost::bad\_lexical\_cast & ) {

204  throw json\_exception("number exceeds integer range");

205  }

206  ss >> n;

207  h\_.number\_long(n);

208  }

209  } else {

210  double x;

211  try {

212  x = boost::lexical\_cast<double>(ss.str());

213  } catch (const boost::bad\_lexical\_cast & ) {

214  throw json\_exception("number exceeds double range");

215  }

216  ss >> x;

217  h\_.number\_double(x);

218  }

219  }

220

221  std::string parse\_string\_chars\_quotation\_mark() {

222  std::stringstream s;

223  while (true) {

224  char c = get\_char();

225  if (c == '"') {

226  return s.str();

227  } else if (c == '\\') {

228  c = get\_char();

229  if (c == '\\' || c == '/' || c == '"') {

230  s << c;

231  } else if (c == 'b') {

232  s << '\b';

233  } else if (c == 'f') {

234  s << '\f';

235  } else if (c == 'n') {

236  s << '\n';

237  } else if (c == 'r') {

238  s << '\r';

239  } else if (c == 't') {

240  s << '\t';

241  } else if (c == 'u') {

242  get\_escaped\_unicode(s);

243  } else {

244  throw json\_exception("expecting legal escape");

245  }

246  continue;

247  } else if (c > 0 && c < 0x20) { // ASCII control characters

248  throw json\_exception("found control character, "

249  "char values less than U+0020 must be \\u escaped");

250  }

251  s << c;

252  }

253  }

254

255  void parse\_true\_literal() {

256  get\_chars("rue");

257  h\_.boolean(true);

258  }

259

260  void parse\_false\_literal() {

261  get\_chars("alse");

262  h\_.boolean(false);

263  }

264

265  void parse\_null\_literal() {

266  get\_chars("ull");

267  h\_.null();

268  }

269

270  void get\_escaped\_unicode(std::stringstream& s) {

271  unsigned int codepoint = get\_int\_as\_hex\_chars();

272  if (!(is\_high\_surrogate(codepoint) || is\_low\_surrogate(codepoint))) {

273  putCodepoint(s,codepoint);

274  } else if (!is\_high\_surrogate(codepoint)) {

275  throw json\_exception("illegal unicode values, "

276  "found low-surrogate, missing high-surrogate");

277  } else {

278  char c = get\_char();

279  if (! (c == '\\'))

280  throw json\_exception("illegal unicode values, "

281  "found high-surrogate, expecting low-surrogate");

282  c = get\_char();

283  if (! (c == 'u'))

284  throw json\_exception("illegal unicode values, "

285  "found high-surrogate, expecting low-surrogate");

286  unsigned int codepoint2 = get\_int\_as\_hex\_chars();

287  unsigned int supplemental

288  = ((codepoint - MIN\_HIGH\_SURROGATE) << 10)

289  + (codepoint2 - MIN\_LOW\_SURROGATE)

290  + MIN\_SUPPLEMENTARY\_CODE\_POINT;

291  putCodepoint(s,supplemental);

292  }

293  }

294

295  unsigned int get\_int\_as\_hex\_chars() {

296  std::stringstream s;

297  s << std::hex;

298  for (int i=0; i<4; i++) {

299  char c = get\_char();

300  if (! ((c >= 'a' && c<= 'f')

301  || (c >= 'A' && c<= 'F')

302  || (c >= '0' && c<= '9')))

303  throw json\_exception("illegal unicode code point");

304  s << c;

305  }

306  unsigned int hex;

307  s >> hex;

308  return hex;

309  }

310

311  void putCodepoint(std::stringstream& s, unsigned int codepoint){

312  if (codepoint <= 0x7f)

313  s.put(codepoint);

314  else if (codepoint <= 0x7ff) {

315  s.put(0xc0 | ((codepoint >> 6) & 0x1f));

316  s.put(0x80 | (codepoint & 0x3f));

317  } else if (codepoint <= 0xffff) {

318  s.put(0xe0 | ((codepoint >> 12) & 0x0f));

319  s.put(0x80 | ((codepoint >> 6) & 0x3f));

320  s.put(0x80 | (codepoint & 0x3f));

321  } else {

322  s.put(0xf0 | ((codepoint >> 18) & 0x07));

323  s.put(0x80 | ((codepoint >> 12) & 0x3f));

324  s.put(0x80 | ((codepoint >> 6) & 0x3f));

325  s.put(0x80 | (codepoint & 0x3f));

326  }

327  }

328

329  void get\_chars(const std::string& s) {

330  for (size\_t i = 0; i < s.size(); ++i) {

331  char c = get\_char();

332  if (c != s[i])

333  throw json\_exception("expecting rest of literal: "

334  + s.substr(i));

335  }

336  }

337

338  void parse\_array\_values\_end\_array() {

339  char c = get\_non\_ws\_char();

340  if (c == ']') return;

341  unget\_char();

342  while (true) {

343  parse\_value();

344  char c = get\_non\_ws\_char();

345  if (c == ']') return;

346  if (c != ',') {

347  throw json\_exception("in array, expecting ] or ,");

348  }

349  c = get\_non\_ws\_char();

350  if (c == ']')

351  throw json\_exception("in array, expecting value");

352  unget\_char();

353  }

354  }

355

356  void parse\_object\_members\_end\_object() {

357  char c = get\_non\_ws\_char();

358  if (c == '}') return;

359  while (true) {

360  // string (key)

361  if (c != '"')

362  throw json\_exception("expecting member key"

363  " or end of object marker (})");

364  std::string key = parse\_string\_chars\_quotation\_mark();

365  h\_.key(key);

366  // name-separator separator

367  c = get\_non\_ws\_char();

368  if (c != ':')

369  throw json\_exception("expecting key-value separator :");

370  // value

371  parse\_value();

372

373  // continuation

374  c = get\_non\_ws\_char();

375  if (c == '}')

376  return;

377  if (c != ',')

378  throw json\_exception("expecting end of object } or separator ,");

379  c = get\_non\_ws\_char();

380  }

381  }

382

383  char get\_char() {

384  char c = in\_.get();

385  if (!in\_.good())

386  throw json\_exception("unexpected end of stream");

387  if (c == '\n') {

388  ++line\_;

389  column\_ = 1;

390  } else {

391  ++column\_;

392  }

393  return c;

394  }

395

396  char get\_non\_ws\_char() {

397  while (true) {

398  char c = get\_char();

399  if (is\_whitespace(c)) continue;

400  return c;

401  }

402  }

403

404  void unget\_char() {

405  in\_.unget();

406  --column\_;

407  }

408

409  Handler& h\_;

410  std::istream& in\_;

411  char next\_char\_;

412  size\_t line\_;

413  size\_t column\_;

414  };

415

416  }

417

428  template <bool Validate\_UTF\_8, typename Handler>

429  void parse(std::istream& in,

430  Handler& handler) {

431  parser<Handler,Validate\_UTF\_8>(handler,in).parse();

432  }

433

444  template <typename Handler>

445  void parse(std::istream& in,

446  Handler& handler) {

447  parse<false>(in,handler);

448  }

449

450  }

451 }

452

453 #endif

json\_error.hpp

in\_

std::istream & in\_

**Definition:** json\_parser.hpp:410

line\_

size\_t line\_

**Definition:** json\_parser.hpp:412

stan::json::parse

void parse(std::istream &in, Handler &handler)

Parse the JSON text represented by the specified input stream, sending events to the specified handle...

**Definition:** json\_parser.hpp:429

h\_

Handler & h\_

**Definition:** json\_parser.hpp:409

next\_char\_

char next\_char\_

**Definition:** json\_parser.hpp:411

column\_

size\_t column\_

**Definition:** json\_parser.hpp:413


---

[ Stan Home Page ]

*© 2011–2014,
Stan Development Team.*
